# Laryngospasm During Anesthesia in Emergency Surgery for Ruptured Ectopic Pregnancy: A Case Report

**DOI:** 10.1002/ccr3.70297

**Published:** 2025-03-10

**Authors:** Banafsheh Mashak, Hawraa Shbeeb, Samaneh Yavari, Mehdi Mirzaee, Reza Saeedinia, Marjan Ghaemi

**Affiliations:** ^1^ School of Medicine Alborz University of Medical Sciences Karaj Iran; ^2^ Department of Obstetrics and Gynecology, Family Health Research Institute, Imam Khomeini Hospital Complex, Vali‐e‐Asr Hospital Tehran University of Medical Sciences Tehran Iran; ^3^ Vali‐E‐Asr Reproductive Health Research Center, Family Health Research Institute, Imam Khomeini Hospital Complex Tehran University of Medical Sciences Tehran Iran

**Keywords:** anesthesia, intubation, laryngospasm, obesity, ruptured ectopic pregnancy

## Abstract

This case report highlights the challenges of managing laryngospasm during emergency surgery for a ruptured ectopic pregnancy. Laryngospasm, a serious complication of anesthesia, causes involuntary laryngeal muscle contractions that obstruct the airway and can lead to hypoxemia, unconsciousness, or death if untreated. A 39‐year‐old obese woman presented with acute abdominal pain and symptoms of a ruptured ectopic pregnancy. During anesthesia induction, she experienced laryngospasm, complicating intubation and requiring the use of a laryngeal mask airway. Treatment with subcutaneous epinephrine, nebulized morphine, and aminophylline successfully restored her airway, allowing the surgery to proceed without further complications. This case emphasizes the importance of being prepared to manage laryngospasm, particularly in high‐risk patients with factors like obesity, emotional stress, or a history of asthma. It highlights the need for anesthesiologists to remain vigilant and respond swiftly to early signs of airway obstruction. The report underscores the value of teamwork and timely intervention in managing emergencies to ensure patient safety and prevent life‐threatening outcomes.

**Summary**

- Laryngospasm, a life‐threatening airway complication during anesthesia, demands rapid intervention and preparedness, particularly in high‐risk patients such as those who are pregnant, obese, or have a history of airway diseases like asthma.
- This case emphasizes the importance of effective airway management, multidisciplinary teamwork, and prompt pharmacological strategies to ensure patient safety during complex emergency surgery.

## 1 Introduction

Emergency intubation is a critical procedure extensively utilized as a life‐saving intervention in severe acute illnesses and injuries that could compromise the patient's airway and ventilation. Despite the importance of prompt diagnosis and treatment to prevent such complications, intubation remains essential. Due to the associated risks, the techniques require skilled professionals, such as anesthesiologists and intensive care unit (ICU) staff [1].

A recognized complication of anesthesia and intubation, laryngospasm is an acute and life‐threatening event characterized by involuntary laryngeal muscle contractions and obstruction of the airway. This condition can last from seconds to several minutes, potentially leading to hypoxia, loss of consciousness, and death. Failure to secure a timely and adequate airway, such as rapid sequence intubation (RSI), can quickly lead to life‐threatening conditions [2].

Obesity significantly increases anesthesia‐related risks, especially among pregnant women, by predisposing them to airway closure during induction. Effective preoperative planning, ensuring appropriate anesthetic depth, and prompt response to complications such as laryngospasm are imperative for enhancing patient outcomes in these high‐risk scenarios [3, 4, 5].

Emergency intubation is a life‐saving procedure crucial for managing severe acute illnesses and injuries that threaten a patient's airway and ventilation. Its success hinges on prompt diagnosis and intervention to prevent complications. However, given the inherent risks, this procedure demands the expertise of highly skilled professionals, including anesthesiologists and ICU staff [1].

Laryngospasm, a well‐documented complication of anesthesia and intubation, is a sudden and potentially fatal event marked by involuntary contraction of the laryngeal muscles, resulting in airway obstruction. This condition, which can persist for seconds to several minutes, poses significant risks, including hypoxia, loss of consciousness, and even death. If a secure airway is not established promptly—such as through RSI—the situation can rapidly escalate to a life‐threatening emergency [2].

The risks associated with anesthesia are further amplified in obese patients, particularly pregnant individuals. Obesity increases the likelihood of airway closure during induction, making these patients more vulnerable to complications like laryngospasm [6]. For such high‐risk cases, careful preoperative planning, maintaining adequate anesthetic depth, and immediate intervention in the event of complications are essential for optimizing patient outcomes [6].

## 2 Case Presentation

### 2.1 Case History/Examination

A 39‐year‐old gravida 2, para 1 (G2P1L1) woman presented to the emergency department at Imam Khomeini Hospital Complex (IKHC) in Tehran with acute onset lower abdominal pain localized to the hypogastric region. She reported a 2‐month history of amenorrhea and was uncertain of the date of her last menstrual period, with no prior antenatal follow‐up. Additional symptoms included dizziness, generalized weakness, and brownish vaginal bleeding persisting for 4 days.

The patient had no history of fever or signs suggestive of pelvic inflammatory disease. On clinical examination, she appeared pale and distressed. Her blood pressure was 105/76 mmHg, and her pulse rate was 110 beats per minute.

Abdominal examination revealed distension and tenderness on both superficial and deep palpation, with clinical signs consistent with peritonitis. Per vaginal examination showed bilateral adnexal fullness and cervical motion tenderness. The patient was clinically obese, with a body mass index of 31, standing 163 cm tall and weighing 83 kg.

Her medical history included hypothyroidism managed with levothyroxine 100 μg daily, and asthma, for which she used a salbutamol inhaler, 200 μg per puff every 4–6 h as needed.

### 2.2 Methods

The patient's vital signs prior to entering the operating room included a heart rate of 110 beats per minute, blood pressure of 105/76 mmHg, oxygen saturation of 98% on pulse oximetry, and urinary output of 150 cc.

Initial investigations revealed a positive urine pregnancy test. A bedside pelvic ultrasound showed no evidence of intrauterine gestation. The endometrium appeared normal in the trilaminar secretory phase with a thickness of 10 mm, while a moderate amount of free fluid was detected in the pouch of Douglas. A 3.5 × 4.5 cm thick‐walled cystic structure was visualized in the right adnexa. Laboratory tests showed a hemoglobin level of 8 g/L and a total white cell count of 6.5 g/L, with normal sodium, potassium, albumin, and coagulation profiles (PT/PTT/INR). Blood grouping and crossmatching for two units were promptly arranged.

The diagnosis of a ruptured ectopic pregnancy was explained to the patient, who provided consent for emergency laparotomy with possible salpingectomy. In the operating room, rapid sequence induction of general anesthesia was performed with propofol (2.5 mg/kg) and succinylcholine (1.5 mg/kg) due to the patient's hypotension (BP 90/60 mmHg), which precluded spinal anesthesia. During induction, the patient experienced significant anxiety and developed laryngospasm, preventing successful intubation. The anesthesiologist managed ventilation using a laryngeal mask airway (LMA).

To address the laryngospasm, 1 mg of epinephrine was administered subcutaneously, while 1 mg of morphine was delivered via nebulization. Additionally, 250 mg of aminophylline in 500 mL normal saline was infused over 20 min.

**FIGURE 1:**
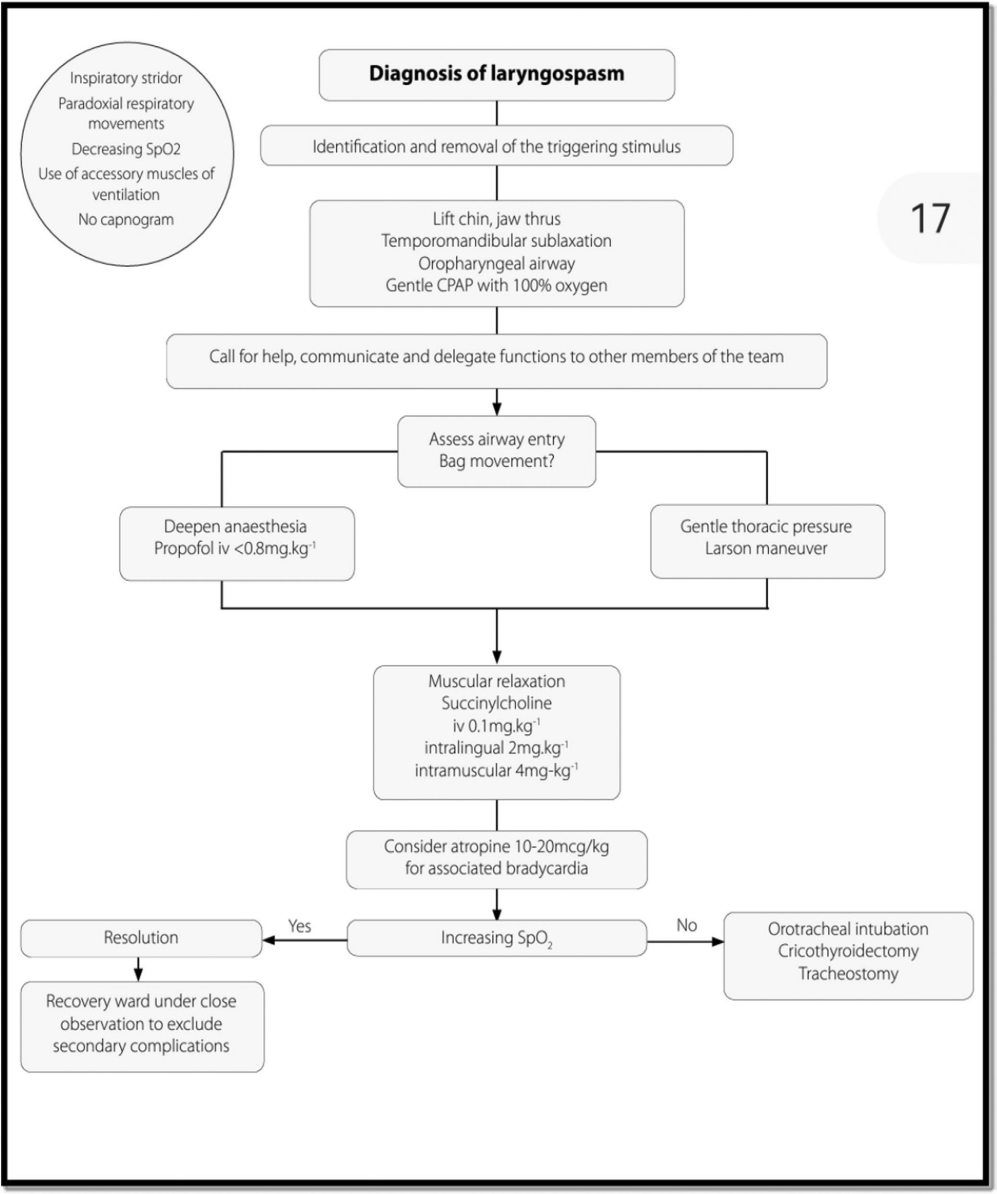
Simplified flowchart for laryngospasm management. From: Update in Anaesthesia/Editors: Christina Lundgren and Victoria Howell/Volume 35 February 2020/ISSN 1353‐4882/Page 17.

Once the patient regained full consciousness and cooperation, spinal anesthesia was administered using a 25‐Gauge spinal needle with 4 cc of marcaine solution. Although the patient reported shortness of breath during the procedure, her symptoms improved by the end of surgery, with notable resolution of hoarseness (Figure 1).

The laparotomy revealed approximately 700 cc of intra‐abdominal blood and 200 cc of clotted blood, which were evacuated. A ruptured right fallopian tube was identified and resected, and the specimen was sent for histopathology. Both ovaries appeared normal. A pelvic drain was inserted, and the histopathology confirmed chorionic villi within the lumen of the right fallopian tube, consistent with a tubal ectopic pregnancy.

During the procedure, the patient received one unit of packed red blood cells. She was subsequently transferred to the ICU for close monitoring over 2 days.

While in the ICU, she remained hemodynamically stable, with oxygen saturation maintained at 6 L O_2_ via face mask. Her chest was clear on auscultation, her abdomen was soft and nontender, and the surgical wound was well‐dressed. The pelvic drain produced approximately 350 cc of hemoserous fluid, and her urine output was adequate. Posttransfusion hemoglobin increased to 9.8 g/L, and her white cell count rose to 11 g/L. Electrolyte levels were balanced, and the patient was managed with antibiotics and NSAIDs.

### 2.3 Conclusions and Results

On postoperative day 3, the patient was transferred from the ICU to the gynecology ward. By 4 days after surgery, she was discharged home in stable condition.

This case underscores the challenges of managing laryngospasm during emergency surgery. The successful resolution of airway obstruction through rapid pharmacological intervention and the use of an LMA highlight the critical importance of prompt and decisive action in emergency settings. It emphasizes the necessity for healthcare providers to maintain vigilance and preparedness to quickly recognize and address airway complications during acute medical crises, ensuring patient safety and optimal outcomes.

## 3 Discussion

Ectopic pregnancy is a significant first‐trimester complication and remains one of the leading causes of maternal mortality, accounting for 9%–13% of pregnancy‐related deaths [7]. While the classic triad of amenorrhea, abdominal pain, and vaginal bleeding is a hallmark presentation, only half of the cases exhibit these symptoms, and clinical manifestations can vary widely [8]. In tubal pregnancies, the absence of a submucosal layer in the fallopian tube allows trophoblasts to invade and erode the muscularis layer, often resulting in tubal rupture, hemorrhage, and shock. Management depends on factors such as hemodynamic stability, BHCG levels, gestational sac size, and fertility goals [9]. In this case, due to significant intra‐abdominal bleeding, hemodynamic instability, and a ruptured ectopic mass, an emergency laparotomy and right salpingectomy were performed. General anesthesia was administered using rapid sequence induction to mitigate airway risks during this critical intervention.

Emergency airway management is essential for ensuring oxygen delivery in patients with respiratory distress, typically achieved through intubation [10]. Clinicians often have a window of 15–30 s to secure an airway before the onset of hypoxia and potential brain injury [11]. RSI, utilized in 80%–85% of emergency department intubations, minimizes the risk of pulmonary aspiration and prevents airway complications from coughing or throat strain during the procedure [12]. RSI involves administering paralytic, sedative, and analgesic medications, followed by airway stabilization with either direct or indirect laryngoscopy [12].

Laryngospasm, though rare, is a serious anesthetic complication characterized by involuntary laryngeal muscle spasms that block the glottis. Symptoms such as severe dyspnea and stridor can rapidly escalate to hypoxemia, unconsciousness, bradycardia, or death if not addressed promptly [13]. Its causes vary and may include anesthesia‐related reactions, emotional distress, obesity, gastroesophageal reflux, tobacco smoke, or upper respiratory infections (URI) [14]. In this case, the patient's pregnancy status, weight, emotional stress, and fear, exacerbated by her first emergency admission, likely triggered the laryngospasm during anesthesia induction. Laryngospasm, an exaggerated airway protective reflex, accounts for approximately 40% of airway obstructions [15]. Timely diagnosis and intervention are critical, as untreated laryngospasm can result in hypoxia, cardiac arrest, and other severe complications. A history of asthma increases the risk of laryngospasm tenfold. In this case, the condition may have been caused by the combination of procedural challenges, such as insufficient anesthesia depth, and physiological factors, such as a history of asthma, obesity, pregnancy, and procedural challenges [15].

Physiological changes during pregnancy, including mucosal swelling, increased vascularity, and reduced functional residual capacity, heighten airway reactivity. Hormonal effects from elevated estrogen and progesterone further narrow the airway, making pregnant patients more susceptible to laryngospasm, particularly under inadequate anesthesia [13, 14]. Stress related to a ruptured ectopic pregnancy can further exacerbate airway sensitivity through heightened vagal reflexes [3, 16].

Obesity independently worsens airway challenges by narrowing the airway and reducing its compliance. Excess fat tissue in the neck and airway contributes to a higher risk of airway collapse and rapid oxygen desaturation during apnea [4, 5]. Obese individuals also exhibit increased laryngeal reactivity, further predisposing them to laryngospasm. In this case, light anesthesia failed to sufficiently suppress airway reflexes, leading to laryngospasm during induction [16, 17].

Rapid sequence induction was chosen to address the patient's unstable condition and low blood pressure. However, insufficient anesthesia depth and airway reactivity combined to trigger laryngospasm. This highlights the need for thorough preanesthetic planning, including the selection of appropriate sedatives and consideration of alternative airway strategies to minimize risks [13, 16, 18].

The successful resolution of laryngospasm in this case, achieved with epinephrine, nebulized morphine, aminophylline, and an LMA, underscores the importance of preparedness in managing airway emergencies in high‐risk patients. This case also illustrates the need for a multidisciplinary approach to address obstetric emergencies involving airway complications [13, 16, 18].

Laryngospasm is caused by a reflex arc that involves the laryngeal nerve, resulting in airway closure to protect against aspiration during swallowing. Paroxysmal laryngospasm, a severe form of this reflex, leads to complete airway closure and is marked by sudden respiratory distress, hoarseness, and stridor. Triggers can include URIs, emotional agitation, or coughing, with laryngospasm contributing to 43% of anesthesia‐related respiratory complications [19]. Management involves removing the underlying stimulus, administering 100% oxygen, and performing airway maneuvers such as jaw thrust or inserting an oropharyngeal airway. Additional treatments may include nebulized lidocaine, propofol, or neuromuscular blockers like succinylcholine if initial efforts fail. Emergency procedures such as cricothyrotomy or tracheostomy are reserved as last‐resort measures [20].

Recognizing risk factors such as obesity, medical history, and surgical conditions is vital for preventing laryngospasm. Ensuring adequate anesthesia depth during intubation is particularly crucial. This case highlights how pregnancy and obesity, combined with anesthesia challenges, exacerbate airway complications, emphasizing the importance of risk assessment, planning, and preparation to ensure patient safety.

## 4 Conclusion

Obesity, pregnancy, coexisting airway conditions, and medical issues like hypothyroidism significantly increase the risk of laryngospasm by altering airway structure and function. Obesity contributes to physical airway obstruction and heightened reactivity, while pregnancy introduces hormonal and anatomical changes that make the airway more prone to spasms. Effective management requires maintaining adequate anesthesia depth, vigilant monitoring, and careful airway planning to minimize these risks. In emergencies, rapid pharmacological intervention and the expertise of skilled anesthesia teams are critical in preventing severe complications. This underscores the importance of readiness, precision, and a multidisciplinary approach in managing airway emergencies in high‐risk patients.

## Author Contributions

**Banafsheh Mashak:** conceptualization. **Hawraa Shbeeb:** conceptualization, writing – original draft, writing – review and editing. **Samaneh Yavari:** methodology. **Mehdi Mirzaee:** validation. **Reza Saeedinia:** data curation. **Marjan Ghaemi:** conceptualization, supervision.

## Ethics Statement

We strictly adhered to the principles of the Declaration of Helsinki throughout the whole study process. Also, this study was approved by the Research and Ethics Committee of Tehran University of Medical Sciences.

## Consent

Written and formal consent for the publication of this case report was obtained from the patient.

## Conflicts of Interest

The authors declare no conflicts of interest.

## Data Availability

Data sharing is not applicable to this article as no datasets were developed or analyzed during the study.
